# Supplementary material for: Electrophysiological correlates of symbolic numerical order processing
Source: PLoS One. 2024 Mar 21;19(3):e0301228. doi: 10.1371/journal.pone.0301228 (PMC10956805; doi:10.1371/journal.pone.0301228)
Supplement: S1 Table — Note that we presented sequences without dashes in the experiment. (DOCX) [file pone.0301228.s001.docx]

|  |  | Sequence | Order | Distance | Direction | Repetitions |
| --- | --- | --- | --- | --- | --- | --- |
| 1 |  | 2–3–4 | ordered | 1 | ascending | 10 |
| 2 |  | 3–4–5 | ordered | 1 | ascending | 10 |
| 3 |  | 4–5–6 | ordered | 1 | ascending | 10 |
| 4 |  | 5–6–7 | ordered | 1 | ascending | 10 |
| 5 |  | 6–7–8 | ordered | 1 | ascending | 10 |
| 6 |  | 7–8–9 | ordered | 1 | ascending | 10 |
| 7 |  | 3–2–1 | ordered | 1 | descending | 10 |
| 8 |  | 4–3–2 | ordered | 1 | descending | 10 |
| 9 |  | 5–4–3 | ordered | 1 | descending | 10 |
| 10 |  | 6–5–4 | ordered | 1 | descending | 10 |
| 11 |  | 7–6–5 | ordered | 1 | descending | 10 |
| 12 |  | 8–7–6 | ordered | 1 | descending | 10 |
| 13 |  | 1–3–5 | ordered | 2 | ascending | 12 |
| 14 |  | 2–4–6 | ordered | 2 | ascending | 12 |
| 15 |  | 3–5–7 | ordered | 2 | ascending | 12 |
| 16 |  | 4–6–8 | ordered | 2 | ascending | 12 |
| 17 |  | 5–7–9 | ordered | 2 | ascending | 12 |
| 18 |  | 5–3–1 | ordered | 2 | descending | 12 |
| 19 |  | 6–4–2 | ordered | 2 | descending | 12 |
| 20 |  | 7–5–3 | ordered | 2 | descending | 12 |
| 21 |  | 8–6–4 | ordered | 2 | descending | 12 |
| 22 |  | 9–7–5 | ordered | 2 | descending | 12 |
| 23 |  | 2–3–1 | unordered | 1 | ascending | 10 |
| 24 |  | 3–4–2 | unordered | 1 | ascending | 10 |
| 25 |  | 4–5–3 | unordered | 1 | ascending | 10 |
| 26 |  | 5–6–4 | unordered | 1 | ascending | 10 |
| 27 |  | 6–7–5 | unordered | 1 | ascending | 10 |
| 28 |  | 7–8–6 | unordered | 1 | ascending | 10 |
| 29 |  | 3–2–4 | unordered | 1 | descending | 10 |
| 30 |  | 4–3–5 | unordered | 1 | descending | 10 |
| 31 |  | 5–4–6 | unordered | 1 | descending | 10 |
| 32 |  | 6–5–7 | unordered | 1 | descending | 10 |
| 33 |  | 7–6–8 | unordered | 1 | descending | 10 |
| 34 |  | 8–7–9 | unordered | 1 | descending | 10 |
| 35 |  | 3–5–1 | unordered | 2 | ascending | 20 |
| 36 |  | 4–6–2 | unordered | 2 | ascending | 20 |
| 37 |  | 5–7–3 | unordered | 2 | ascending | 20 |
| 38 |  | 5–3–7 | unordered | 2 | descending | 20 |
| 39 |  | 6–4–8 | unordered | 2 | descending | 20 |
| 40 |  | 7–5–9 | unordered | 2 | descending | 20 |
