# Supplementary material for: Electrophysiological correlates of symbolic numerical order processing
Source: PLoS One. 2024 Mar 21;19(3):e0301228. doi: 10.1371/journal.pone.0301228 (PMC10956805; doi:10.1371/journal.pone.0301228)
Supplement: S2 Table — Note that all model terms involving the factor “group” are not significant. The degrees of freedom for the F statistic are 1 and 71 for all terms. (DOCX) [file pone.0301228.s002.docx]

|  | MSE | *F* | *η*^2^ | *p* |
| --- | --- | --- | --- | --- |
| group | 0.208916 | 1.8421 | 0.024418 | .1790 |
| order | 0.004517 | 192.0847 | 0.053410 | < .001 |
| group:order | 0.004517 | 0.5408 | 0.000159 | .4645 |
| distance | 0.001933 | 99.3152 | 0.012332 | < .001 |
| group:distance | 0.001933 | 1.1479 | 0.000144 | .2876 |
| order:distance | 0.001198 | 186.1277 | 0.014290 | < .001 |
| group:order:distance | 0.001198 | 0.1503 | 0.000012 | .6994 |
