# Supplementary material for: Electrophysiological correlates of symbolic numerical order processing
Source: PLoS One. 2024 Mar 21;19(3):e0301228. doi: 10.1371/journal.pone.0301228 (PMC10956805; doi:10.1371/journal.pone.0301228)
Supplement: S3 Table — Note that all model terms involving the factor “group” are not significant. The degrees of freedom for the F statistic are 1 and 71 for all terms. (DOCX) [file pone.0301228.s003.docx]

|  | MSE | *F* | *η*^2^ | *p* |
| --- | --- | --- | --- | --- |
| group | 2.49320 | 0.0235 | 0.000183 | .8785 |
| order | 0.69051 | 18.6655 | 0.038599 | < .001 |
| group:order | 0.69051 | 1.4639 | 0.003139 | .2303 |
| distance | 0.58621 | 32.9287 | 0.056720 | < .001 |
| group:distance | 0.58621 | 0.9400 | 0.001714 | .3356 |
| order:distance | 0.75151 | 60.3567 | 0.123802 | < .001 |
| group:order:distance | 0.75151 | 0.1943 | 0.000455 | .6607 |
