# Supplementary figures and images for: Electrophysiological correlates of symbolic numerical order processing
Source: PLoS One. 2024 Mar 21;19(3):e0301228. doi: 10.1371/journal.pone.0301228 (PMC10956805; doi:10.1371/journal.pone.0301228)

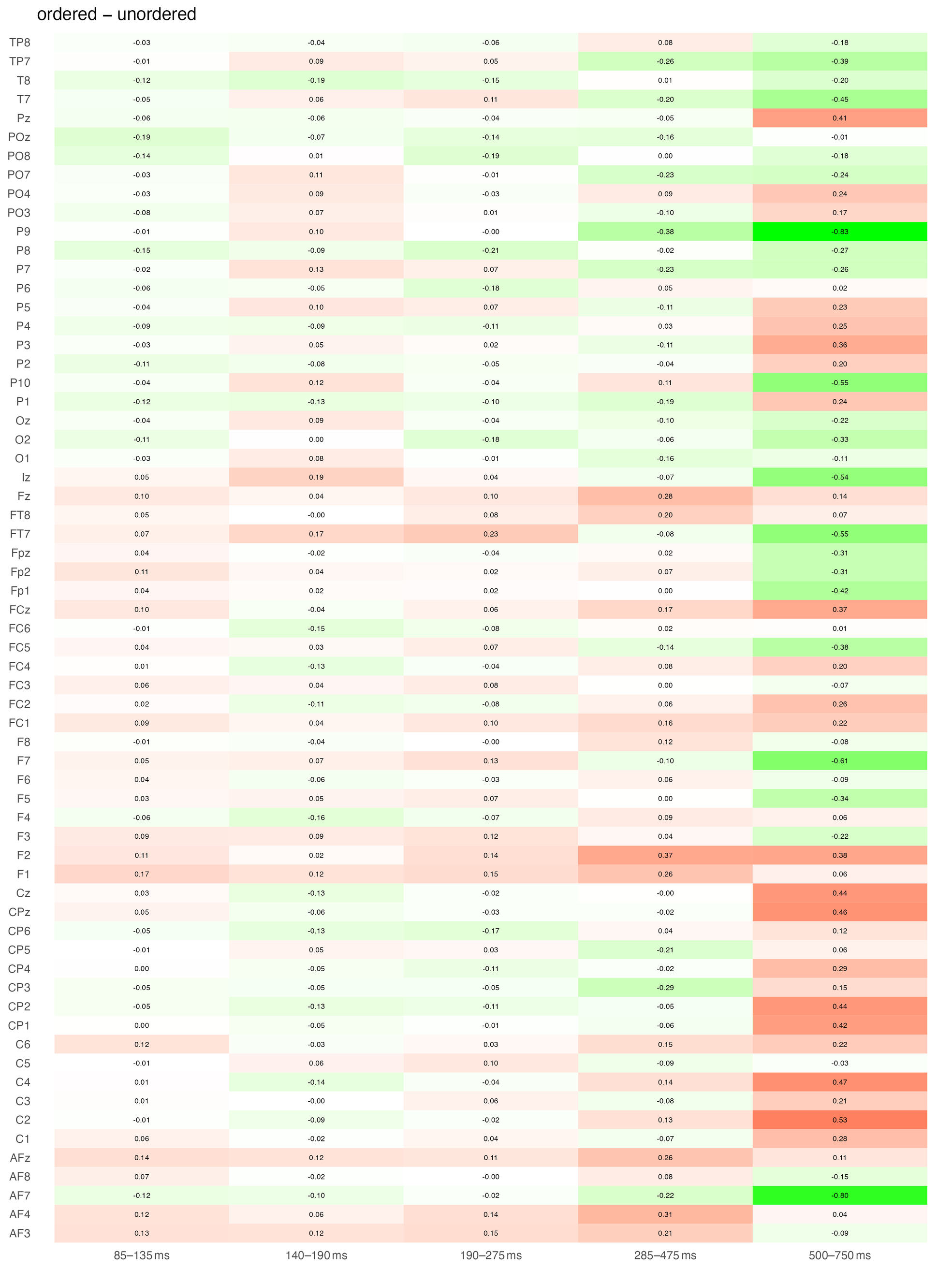

Supplement: S1 Fig — Green indicates negative differences, red indicates positive differences. (TIF) [file pone.0301228.s004.tif]

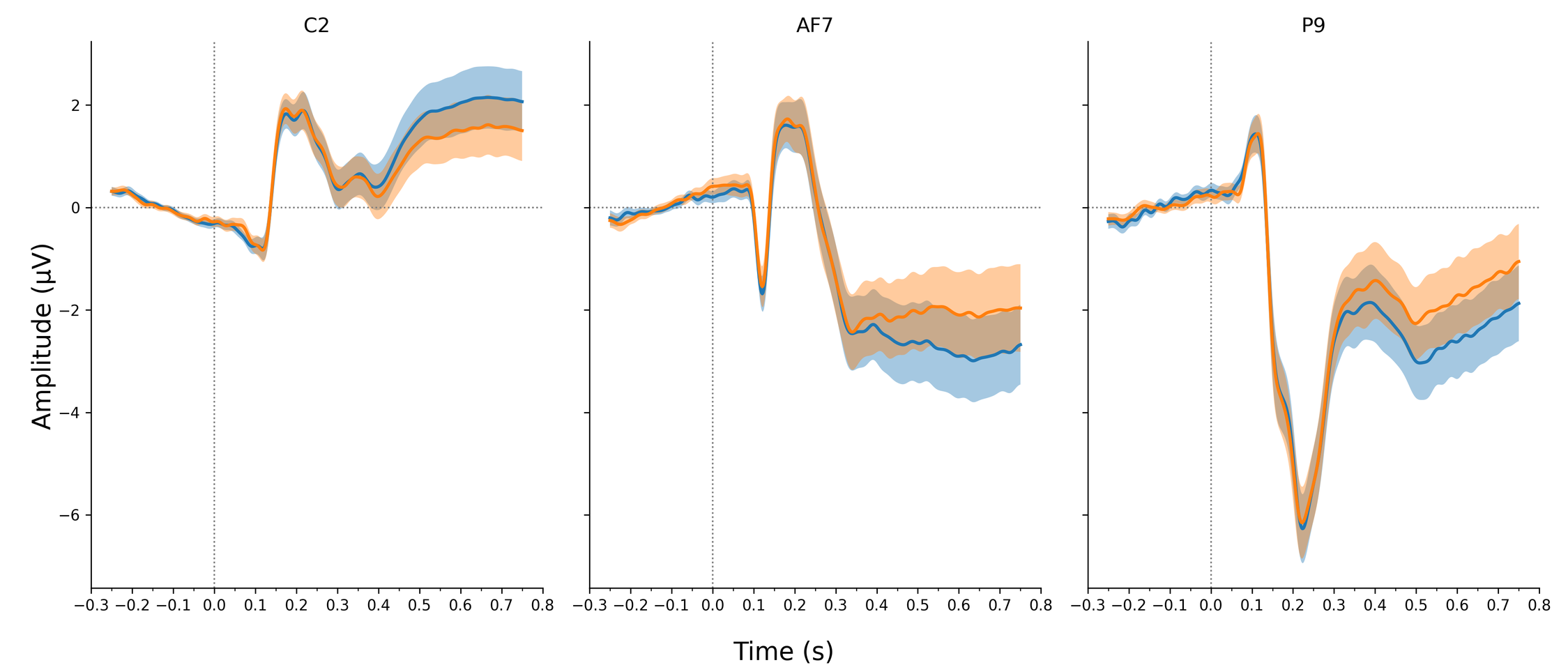

Supplement: S2 Fig — Ribbons indicate 95% confidence intervals. (TIF) [file pone.0301228.s005.tif]

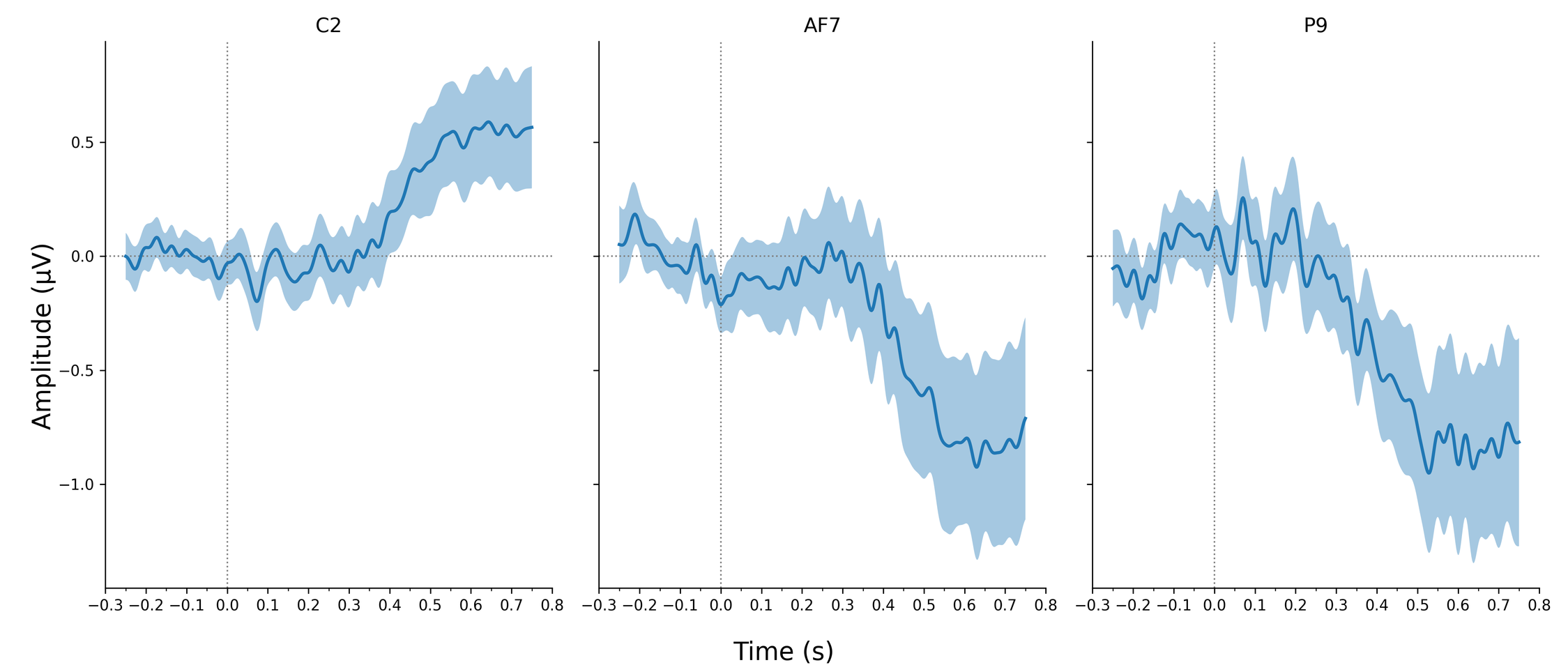

Supplement: S3 Fig — Ribbons indicate 95% confidence intervals. (TIF) [file pone.0301228.s006.tif]

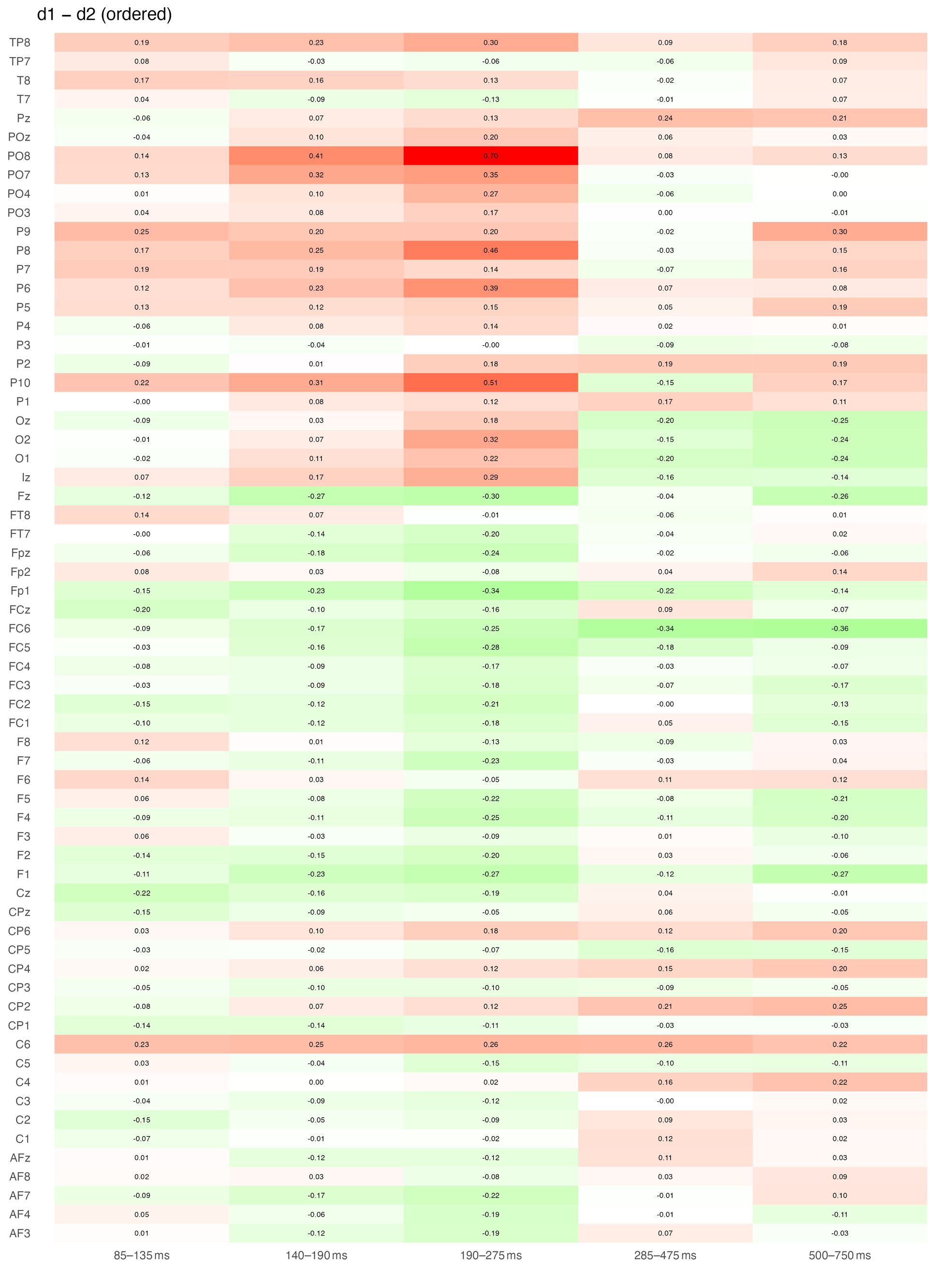

Supplement: S4 Fig — Green indicates negative differences, red indicates positive differences. (TIF) [file pone.0301228.s007.tif]

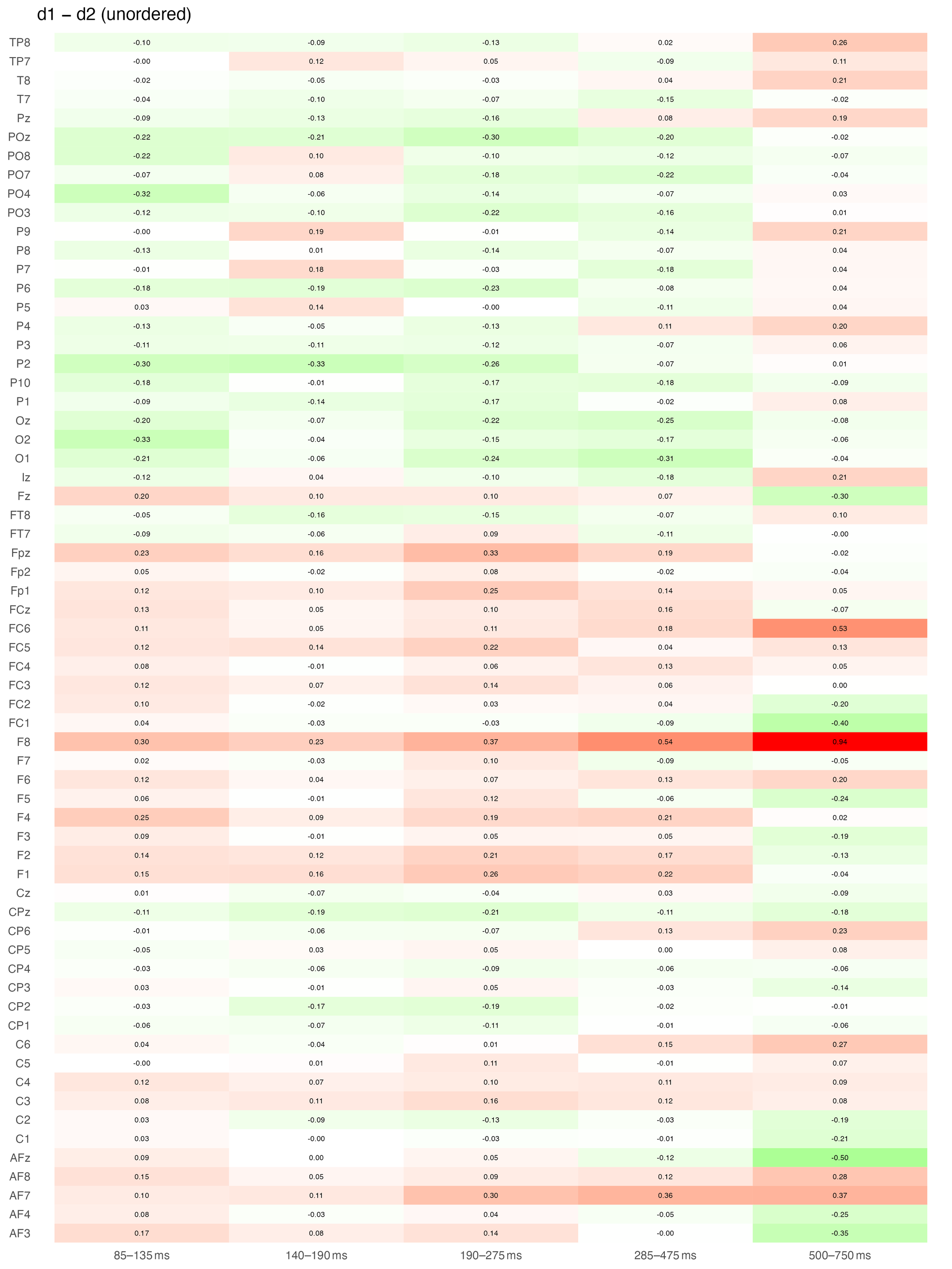

Supplement: S5 Fig — Green indicates negative differences, red indicates positive differences. (TIF) [file pone.0301228.s008.tif]
